# The LRRC8-mediated volume-regulated anion channel is altered in glaucoma

**DOI:** 10.1038/s41598-019-41524-3

**Published:** 2019-04-01

**Authors:** Xavier Gasull, Marta Castany, Aida Castellanos, Mikel Rezola, Alba Andrés-Bilbé, Maria Isabel Canut, Raúl Estévez, Teresa Borrás, Núria Comes

**Affiliations:** 1 Neurophysiology Laboratory, Physiology Unit, Department of Biomedicine, Medical School, University of Barcelona, Casanova 143, E-08036 Barcelona, Spain; 2 Institute of Neurosciences, University of Barcelona, Barcelona, Spain; 3 Institut d’Investigacions Biomèdiques August Pi i Sunyer (IDIBAPS), Barcelona, Spain; 4 Department of Ophthalmology, Hospital Vall d’Hebron, Passeig de la Vall d’Hebron 119-129, E-08035 Barcelona, Spain; 5 Institut Universitari Barraquer, Muntaner 314, E-08021 Barcelona, Spain; 6 Unitat de Fisiologia, Departament de Ciències Fisiològiques, University of Barcelona-IDIBELL, Centro de Investigación en red de enfermedades raras (CIBERER), SCIII., Feixa Llarga s/n, L’Hospitalet de Llobregat, E-08907 Spain; 7 Department of Ophthalmology, University of North Carolina, School of Medicine, 6109 Neuroscience Research Building, 105 Mason Farm Road, Chapel Hill, 27599-7041 North Carolina USA

## Abstract

Regulation of cellular volume is an essential process to balance volume changes during cell proliferation and migration or when intracellular osmolality increases due to transepithelial transport. We previously characterized the key role of volume-regulated anion channels (VRAC) in the modulation of the volume of trabecular meshwork (TM) cells and, in turn, the aqueous humour (AH) outflow from the eye. The balance between the secretion and the drainage of AH determines the intraocular pressure (IOP) that is the major casual risk factor for glaucoma. Glaucoma is an ocular disease that causes irreversible blindness due to the degeneration of retinal ganglion cells. The recent identification of Leucine-Rich Repeat-Containing 8 (LRRC8A-E) proteins as the molecular components of VRAC opens the field to elucidate their function in the physiology of TM and glaucoma. Human TM cells derived from non-glaucomatous donors and from open-angle glaucoma patients were used to determine the expression and the functional activity of LRRC8-mediated channels. Expression levels of LRRC8A-E subunits were decreased in HTM glaucomatous cells compared to normotensive HTM cells. Consequently, the activity of VRAC currents and volume regulation of TM cells were significantly affected. Impaired cell volume regulation will likely contribute to altered aqueous outflow and intraocular pressure.

## Introduction

Glaucoma is a chronic disease in which retinal ganglion cell degeneration leads to an optic nerve damage that results in visual field loss. This group of optic neuropathies represent a significant cause of blindness worldwide^1^. Although the precise molecular mechanisms leading to glaucoma are poorly understood it is known that intraocular pressure (IOP) is the main risk factor for glaucoma development. IOP is maintained through a balance between the amount of aqueous humour (AH) produced in the ciliary processes and the AH drainage. In humans, the main outflow route of AH outflow consists of the trabecular meshwork (TM) tissue and Schlemm’s canal (SC). TM cells actively regulate the drainage of AH, thereby maintaining a physiological intraocular pressure (IOP)^2^. Although the bases for AH outflow regulation are still unknown, different cellular mechanisms have been associated to the trabecular meshwork physiology including composition and remodelling of TM extracellular matrix^2^, contraction / relaxation^3^ and volume regulation of trabecular cells^4–6^, among others. When functionality of TM is impaired, an increased resistance to the eye fluid results in ocular hypertension and glaucoma^7^.

Cell volume regulation is crucial for cell division, migration and death^8^. Swollen cells recover their initial volume by the transport of solutes (especially K^+^ and Cl^−^), organic osmolytes and water through the plasma membrane (PM); this cellular mechanism is known as regulatory volume decrease (RVD)^9^. TM cells possess a RVD^5,6^ mediated at least by the Na^+^/H^+^ antiport^5^, the Na^+^-K^+^−2Cl^−^ co-transporter^5,10^, the large-conductance calcium activated potassium channel (BKCa) and the volume-regulated anion channel (VRAC)^5,6^. Volume of trabecular cells influence aqueous outflow since compounds that induce TM cell swelling reduce outflow facility and compounds known to shrink trabecular cells increase it^4–6,11^. We and others have described how BKCa and VRAC ion channels can modulate aqueous outflow facility as a consequence of regulating the volume of trabecular cells^5,6,12^. Besides volume regulation, VRAC participates in cellular proliferation, migration, apoptosis and release of glutamate^13^. It is widely known that VRAC mediates the ubiquitous swelling-activated chloride current (ICl_swell_)^9^. The well-described electrophysiological properties of VRAC are outwardly rectification, inactivation at large depolarized potentials and iodide over chloride selectivity^13^ while its molecular identity has been highly controversial for decades^14^.

Leucine-Rich Repeat-Containing 8A (LRRC8A) has been identified in a genome-wide loss of function screening^15,16^ as a protein indispensable for the VRAC activity. Specific knockdown of LRRC8A dramatically reduces swelling-activated iodide influx, release of taurine and glutamate^15–17^ and the ability to modulate cell volume^15,16^. LRRC8A was cloned from a patient with congenital agammaglobulinemia, a disease characterized by a deficiency of circulating B lymphocytes^18^. LRRC8A is the first member of protein family that contains five different members (LRRC8A-LRRC8E). The traffic of the LRRC8B-LRRC8E subunits to the cell surface depends on the co-expression with LRRC8A^16^. LRRC8 proteins contain a leucine-rich repeat domain at the C-terminus^19^ and it has been proposed to have four transmembrane segments^20^ and a similar topology to pannexins^21^. Because LRRC8A overexpression causes an unexpected suppression of endogenous VRAC currents^16,22^, it has been speculated that a very specific stoichiometry of LRRC8 subunits is required to form functional VRAC. In this sense, VRAC appear to require an heteromeric composition with at least one main subunit LRRC8A and at least another LRRC8 family member^15,16^. Recent reports suggest that functional channels may operate as hexamers^21,23,24^ containing at least three different LRRC8s^25^. Notably, different combinations of LRRC8B-E plus LRRC8A yield VRAC currents with different inactivation kinetics, rectification and single-channel conductance^22^. As pointed by mutations in the essential subunit LRRC8A^22^, the composition of VRAC determines the channel permeability profile. In addition to transport neurotransmitters and neuromodulators^25^, LRRC8A and LRRC8D subunits have been involved in anticancer drug uptake and LRRC8D is associated to blasticidin transport^26^. In this context, and because VRAC is implicated in aqueous outflow^5,6^, the recent attribution of this activity to LRRC8 proteins paves the way to determine their potential role in TM physiology and glaucoma.

## Methods

### Human trabecular meshwork cell lines

Human transformed TM cells from a non-glaucomatous donor (HTM-5) and from an individual affected by primary open-angle glaucoma (POAG) (HTM-3) were kindly obtained from Abbot Clark (University of North Texas - Health Science Center, TX, USA)^27^. Cells were maintained in Dulbecco’s Modified Eagle’s Medium (DMEM) supplemented with 10% fetal bovine serum (FBS), 100 mg/mL L-glutamine, 100 U.I./mL penicillin and 100 μg/mL streptomycin. Cell passages were performed using Trypsin-EDTA. Reagents were obtained from Sigma-Aldrich. As an *in vitro* model of steroid-induced glaucoma, dexamethasone (DEX; 100 nM in DMEM) or vehicle (0.1% ethanol) was added to control HTM-5 cells every other day during 3 days or 7 days. At designated time points, cells were collected and analysed for mRNA (LRRC8A-LRRC8E and myocilin (MYOC)) and protein (LRRC8A and MYOC).

### Human trabecular meshwork primary cells

Trabecular meshwork (TM) cultured cells from three normotensive non-glaucomatous human donors were kindly provided by D.W. Stamer (Duke University Eye Center, Durham, NC, USA)^28,29^ and T. Borrás (University of North Carolina, Chapel Hill, NC, USA). Cell cultures were maintained in low glucose DMEM supplemented with 10% FBS, 100 mg/mL L-glutamine, 100 U.I./mL penicillin and 100 μg/mL streptomycin in a humidified 5% CO_2_ atmosphere at 37 °C. After confluence, TM cells were passaged using Trypsin-EDTA. Glaucomatous HTM cells from five patients were obtained using the explant method from trabeculectomies performed by Dr. M. I. Canut (Institut Universitari Barraquer, Barcelona, Spain) as described previously^30^ (Table 1). Briefly, a small piece of TM was removed by a punch trabeculectomy and was kept in PBS at 4 °C for not more than 24 h. Human samples were handled in accordance with the principles expressed in the Declaration of Helsinki. TM tissue was carefully attached to the bottom of a 35-mm dish and covered with a drop of DMEM supplemented with 20% FBS, 100 mg/mL L-glutamine, 100 U.I./mL penicillin and 100 μg/mL streptomycin under a glass coverslip. Dishes were incubated in a humidified 5% CO_2_ atmosphere at 37 °C and medium was changed every other day. Cells were allowed to grow from the explant for a period of 4 weeks. Once confluent, TM cells were passed to a T-25 flask, labelled as passage 1, and were maintained in the same medium with 10% FBS. Cells were used at passages 3 to 7 and changed into media containing 1% FBS for one week prior to experimentation. All reagents were obtained from Sigma (Madrid, Spain).

**Table 1:** Summary of Individual Parameters.

Individual	Age (years)	Race	Gender	Number
1	51	Caucasian	Male	Normotensive
2	58	African American	Male	Normotensive
3	45	African American	Male	Normotensive
4	48	Caucasian	Female	Secondary open-angle glaucoma
5	70	Caucasian	Male	Primary open-angle glaucoma
6	78	African	Male	Primary open-angle glaucoma
7	78	Caucasian	Female	Primary open-angle glaucoma
8	67	Caucasian	Male	Primary open-angle glaucoma

### Patch clamp procedures

Patch clamp experiments were performed at 22–23 °C using the whole-cell configuration without any leak subtraction. Electrodes were fabricated in a micropipette puller P-97 (Sutter instruments) with a resistance of around 3 MΩ. A Ag/AgCl ground electrode mounted in a 3 M KCl agar bridge was employed. The setup consists of an inverted microscope (Axiovert 35 M Zeiss), an amplifier (Axopatch 200B, Molecular Devices) and the pClamp 10 software (Molecular Devices). Data analysis was performed with Clampfit 10 (Molecular Devices) and Prism 4 (GraphPad Software, Inc.). Series resistance was kept at <15 MΩ and compensated

at 70–80%. Tamoxifen was purchased from Sigma-Aldrich (Madrid). Swelling-activated Cl^−^ currents were recorded by depolarizing pulses from −100 mV to +100 mV in 20 mV voltage steps applied every 5 seconds from a 0 mV holding potential, using an intracellular pipette solution containing (in mM): 150 NMDGCl, 1.2 MgCl_2_, 1.0 EGTA, 2.0 ATP, 0.5 GTP, 10 Hepes; pH 7.35 adjusted with tris-base (303 mOsm/Kg). The isotonic bath solution contained (in mM): 150 NMDGCl, 0.5 MgCl_2_, 1.3 CaCl_2_, 10 Hepes and 20 D-Sorbitol; pH 7.35 adjusted with tris-base (301.5 ± 3 mOsm/Kg). The hyposmotic solution contained (in mM): 100 NMDGCl, 0.5 MgCl_2_, 1.3 CaCl_2_, 10 Hepes; pH 7.35 adjusted with tris-base (211 ± 3 mOsm/Kg, −30%). To evoke whole-cell outward K^+^ currents, cells were clamped at −60 mV and depolarizing pulses from −100 to +90 mV were applied in 10-mV steps. For clarity only pulses from 0 to +40 mV are shown. The pipette solution was (in mM): 140 KCl, 2.1 CaCl_2_, 2.5 MgCl_2_, 5 EGTA, and 10 HEPES, pH 7.3 with 0.5 M KOH. The bath solution was (in mM): 145 NaCl, 5 KCl, 2 CaCl_2_, 2 MgCl_2_, 10 HEPES and 5 glucose, pH 7.4 with NaOH. Osmolalities were measured using a Vapro osmometer (Wescor) and adjusted with sorbitol.

### RNA extraction and reverse transcription reaction

RNA extraction was conducted with the RNeasy^®^ Mini Kit from QIAGEN (Valencia). Reverse transcription reactions were carried out with spectrophotometrically measured RNA (5 μg; Nanodrop, ThermoFisher Scientific) using the Cloned AMV First-Strand mRNA Synthesis Kit (Invitrogen, ThermoFisher Scientific) according the manufacturer’s recommendations^31^.

### Gene primers

Primers sets for LRRC8A-LRRC8E subunits designed by T. Jentsch and collaborators were used^16^. Primers for glaucoma-related genes (MYOC, ELAM-1, MGP), Na^+^-K^+^-2Cl^−^ cotransporter (NKCC1), BKCa channel (KCNMA1) as well as the ribosomal 18 S RNA were designed using the Primer Designing Tool of the National Center for Biotechnology Information (NCBI) and purchased from Invitrogen (ThermoFisher Scientific) (Table 2). Target specificity was obtained by performing Basic Local Alignment Search Tool (BLAST) comparisons against the entire nucleotide database (http://www.ncbi.nlm.nih.gov).

**Table 2:** Primer sequences, annealing conditions and amplicon lengths for each amplified gene.

Gene		PCR primer sequence	Annealing (°C)	Product size (bp)
LRRC8A	Forward	5′-GGGTTGAACCATGATTCCGGTGAC-3′	54	133
	Reverse	5′-GAAGACGGCAATCATCAGCATGAC-3′		
LRRC8B	Forward	5′-ACCTGGATGGCCCACAGGTAATAG-3′	56	126
	Reverse	5′-ATGCTGGTCAACTGGAACCTCTGC-3′		
LRRC8C	Forward	5′-ACAAGCCATGAGCAGCGAC-3′	52	132
	Reverse	5′-GGAATCATGTTTCTCCGGGC-3′		
LRRC8D	Forward	5′-ATGGAGGAGTGAAGTCTCCTGTCG-3′	54	126
	Reverse	5′-CTTCCGCAAGGGTAAACATTCCTG-3′		
LRRC8E	Forward	5′-ACCGTGGCCATGCTCATGATTG-3′	54	62
	Reverse	5′-ATCTTGTCCTGTGTCACCTGGAG-3′		
ELAM-1	Forward	5′-CCCATGGAACACAACCTGTACATT-3′	54	118
	Reverse	5′-AGCTTTACACGTTGGCTTCTCGTT-3′		
MGP	Forward	5′-CGCCTTAGCGGTAGTAACTTTGT-3′	48	371
	Reverse	5′-TTTTCTTCCCTCAGTCTCATTTG-3′		
MYOC	Forward	5′-AGCACGGGTGCTGTGGTGTAC-3′	49	338
	Reverse	5′-AAGGTGCCACAGATGATGAA-3′		
KCNMA1	Forward	5′-TACTTCAATGACAATATCCTCACCCT-3′	50	210
	Reverse	5′-TTAGGGGATGGTGGTTGTTATGGT-3′		
NKCC1	Forward	5′-CAAGACATACCGGCAGATCAG-3′	50	109
	Reverse	5′-CGAAAAGGTGCTGTGTCTAGT-3′		
18S	Forward	5′-GGCGCCCCCTCGATGCTCTTAG-3′	60	Internal reference for RT-PCR
	Reverse	5′-GCTCGGGCCTGCTTTGAACACTCT-3′		

### Standard PCR reaction and sequencing

PCRs were carried out in a DNA thermal cycler (Eppendorf) to verify the size of amplified products. The protocol was 95 °C for 30 seconds and cycles of 95 °C for 30 seconds, variable annealing temperature for 60 seconds, 68 °C for 60 seconds, and a final extension at 68 °C for 5 min. Amplification of ELAM-1, MGP and MYOC genes was verified by sequentiation (Stab Vida) using the http://www.ncbi.nlm.nih.gov database.

### Relative quantitative real-time PCR

Normalized mRNA quantification was performed by relative quantitative real-time PCR technology with Fast SYBR^TM^ Green Master Mix using an ABI Prism^®^ 7500 Fast Real-Time PCR System and the 7500 System SDS software (Applied Biosystems, ThermoFisher Scientific). Fold Changes were calculated by the formula 2^ΔΔCT^^31^. Melting curves were carried out to determine melting temperatures in order to check the specificity of the amplified products. All experiments were performed by triplicate.

### Perfused human anterior segment organ cultures

Non-glaucomatous human eyes were obtained from national eye banks following signed consent of the patients’ families. All procedures were in accordance with the Tenets of the Declaration of Helsinki. After dissection, pairs of ocular anterior segments were perfused at a constant flow and pressure (P) was monitored as described previously^31^. After a stable baseline of 24 h, a ΔP of 31.96 ± 3.25 mmHg was applied to one eye and the contralateral eye was maintained at physiological pressure (≈15 mmHg). After 7 d, anterior segments were immersed in RNAlater (Ambion) to accomplish dissection of TM tissue and RNA extraction^31^.

### RNA microarrays

RNAs from perfused anterior segments were hybridized to Human Genome U95Av2 or U133Plus2.0 GeneChips (Affymetrix, ThermoFisher Scientific) at the University of North Carolina at Chapel Hill Functional Genomics Core Facility. Affymetrix GeneChip Microarray Suite 5.0 (Affymetrix, ThermoFisher Scientific) and 7.3.1 GeneSpring GX Expression Analysis software (Agilent Technologies) were used to analyse differential gene expression between high P and control P TM tissues^31^.

### Total protein extraction and western blotting

Cultured HTM cells were lysed with a standard 1% Triton X-100 buffer supplemented with 1 μg/ml aprotinin, 1 μg/ml leupeptin, 1 μg/ml pepstatin and 1 mM phenylmethylsulfonyl fluoride (PMSF) as protease inhibitors. Pierce^TM^ BCA Protein Assay Kit (Thermo Scientific) was used to quantify total protein in order to analyse the same amount of HTM-5/HTM-3 paired extracts by western blot (WB). PVDF membranes were incubated with LRRC8A polyclonal antibody (1:1000, Bethyl Laboratories), β-actin polyclonal antibody (1:5000, Sigma-Aldrich) or MYOC monoclonal antibody (1:1000, Santa Cruz Biotechnology) and secondary horseradish peroxidase (HRP)-conjugated goat anti-rabbit (1:10000) or goat anti-mouse (1:2000) antibodies (Jackson ImmunoResearch Laboratories). Immunoreactive bands were captured using the WesternBright^TM^ Quantum detection kit for HRP (Advansta) and visualized by chemiluminescence on Carestream^®^ Kodak BioMax MR films (Sigma-Aldrich) or with a LAS-3000 Mini gel Imaging System (Fujifilm). All experiments were done by triplicate.

### Biotinylation of cell surface proteins

HTM cell-surface biotinylation was carried out using the EZ-Link^®^ Sulfo-NHS-LC-Biotin (sulfosuccinimidyl-6-[biotin-amido]hexanoate) reagent (Thermo Scientific). *N*-Hydroxysuccinimide (NHS) esters of biotin react with primary amines of lysine aminoacidic residues exposed extracellularly. The cross-linking reaction was quenched with 0.1 M Glycine. Cells were scrapped with 1% Triton X-100 buffer with the same cocktail of protease inhibitors than for total protein extraction. When protein content was extracted from the supernatant, a fraction of 10% (in volume) was stored at −20 °C as starting materials. To isolate the labelled proteins, cell lysates were purified on Neutravidin^®^ Agarose Resins (Thermo Scientific) and eluted with 1X Laemmli SDS loading buffer. Starting materials and biotinylated fractions were analysed by western blotting to quantify the LRRC8A levels. Experiments were performed by triplicate.

### Measurements of cell volume with calcein fluorescence

Fluorescence intensity of intracellular calcein was used to quantify changes in cell volume^32^. HTM cells were loaded with 0.5 μM calcein-AM (Calbiochem, Merck KGaA) at 37 °C for 2 min. After acute harvesting, coverslips were mounted on an inverted, epi-fluorescence microscope Olympus IX70 (Olympus). Using a xenon lamp and a monochromator Polychrome IV (Till Photonics), calcein was excited every 20 sec at 488 nm and light emitted at 520 nm was detected with a digital charge-coupled device CCD camera and the AquaCosmos 2.5 Software (Hamamatsu). Isotonic solution contained (in mM): 125 NaCl, 5.5 KCl, 1.3 CaCl_2_, 1.25 MgCl_2_, 20 Hepes, 10 dextrose, 20 BDM; pH 7.4 adjusted with NaOH (292 mOsm/Kg). Hypotonic solution was (in mM): 80 NaCl, 5.5 KCl, 1.3 CaCl_2_, 1.25 MgCl_2_, 20 Hepes, 10 dextrose, 20 BDM; pH 7.4 adjusted with NaOH (213 mOsm/Kg, 27%). Osmotic behaviour of each cell was analysed by applying 14% hypotonic (251 mOsm/Kg) / hypertonic (333 mOsm/Kg) before experiments. Only cells with an osmotic behaviour were analysed to determine the RVD mechanism^12^.

### Data analysis

Results are presented as mean ± SEM. Data were analysed with paired or unpaired Student’s t-tests. Entire I-V curves of different experimental conditions were analysed with two-way analysis of variance (ANOVA) plus Bonferroni post-tests. Prism 4.0 software was used (GraphPad Software). p < 0.05 was considered statistically significant.

## Results

### Validation of both glaucomatous (HTM-3) and control (HTM-5) trabecular meshwork cells at a molecular level

**Figure 1:**
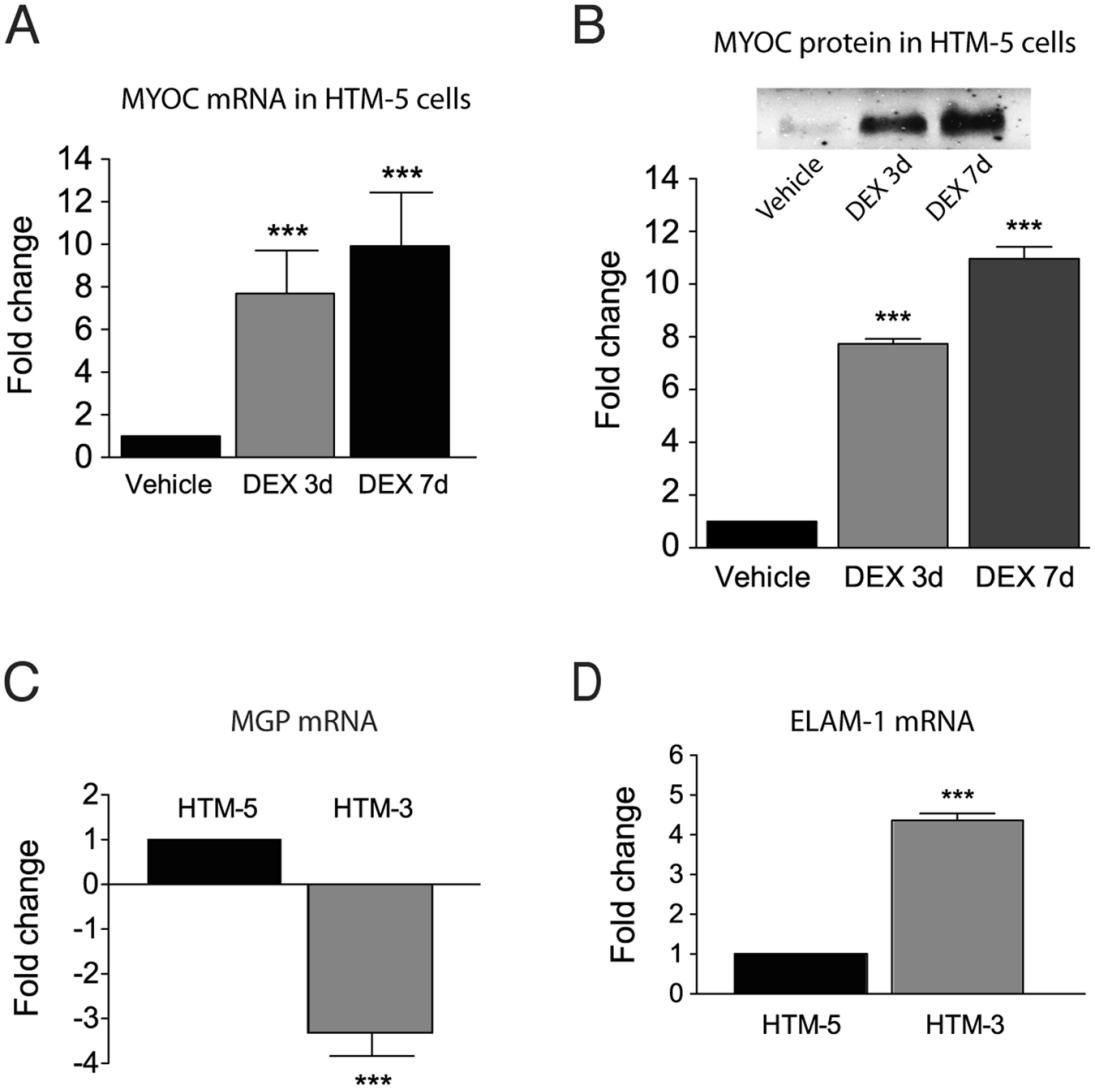
Validation of HTM-5 and HTM-3 cell lines. (**A**) Induction of Myocilin (MYOC) mRNA in HTM-5 cells treated with dexamethasone (DEX, 100 nM) at two different time points (3 days and 7 days) compared to HTM-5 cells treated with vehicle (0.1% ethanol). Values are the mean ± SEM of n = 8 cells (***p < 0.001 DEX-treated cells vs. vehicle, Student’s t-test). **(B)** MYOC protein quantified in HTM-5 extracts treated with vehicle, DEX 3 days and DEX 7 days. MYOC was significantly increased by DEX. Values are mean ± SEM of n = 8 cells (***p < 0.001 DEX-treated cells vs vehicle, Student’s t-test). *Inset*: Representative western blot showing MYOC in HTM-5 cells. A full-length blot is presented in Supplementary Fig. 1B. **(C)** mRNA expression of Matrix Gla Protein (MGP) and **(D)** Endothelial-Leukocyte Adhesion Molecule-1 (ELAM-1) in glaucomatous HTM-3 cells normalized to HTM-5 control cells. MGP was significantly down-regulated and ELAM-1 was significantly up-regulated in HTM-3 compared to HTM-5, as it has been described in glaucoma. Values are mean ± SEM of n = 3 cells (***p < 0.001 HTM-3 versus HTM-5, Student’s t-test).

Trabecular meshwork cell lines from a non-glaucomatous donor (HTM-5) and from a primary open-angle glaucoma patient (POAG) (HTM-3) were used^27^. Prior to undertaking experiments, cell types were validated by quantifying Myocilin (MYOC) mRNA/protein in control HTM-5 cells treated with Dexamethasone (DEX) and the expression levels of glaucoma-related genes in glaucomatous HTM-3 cells. Glucocorticoids induce ocular hypertension^33^ and many studies have shown that induction of MYOC by the glucocorticoid DEX is specific to the TM tissue. HTM-5 cells were treated either with DEX (100 nM in 0.1% ethanol) or vehicle (0.1% ethanol) for 3 or 7 days. MYOC showed low expression in vehicle-treated HTM-5 cells but its expression was significantly increased after 3 or 7 days of DEX exposure (7.69 ± 2.15-fold and 9.92 ± 2.68-fold, respectively, n = 3, p < 0.001) (Fig. 1A). Similarly, DEX increased MYOC protein by 7.74 ± 0.19-fold (3 days) and 10.97 ± 0.45-fold (7 days) in comparison to vehicle-treated cells (n = 3, p < 0.001) (Fig. 1B). To assess whether specific alterations described in glaucoma were present in the HTM-3 cells, we evaluated the expression of Matrix Gla Protein (MGP) and Endothelial-Leukocyte Adhesion Molecule 1 (ELAM-1). Glaucomatous cells present a lower expression of MGP, a gene encoding a protein that acts as an inhibitor of vascular mineralization^30,34^. Accordingly, we found a decreased expression of MGP in HTM-3 compared to HTM-5 cells (−3.32 ± 0.52-fold; n = 3, p < 0.001; Fig. 1C). ELAM-1, which encodes a secreted glycoprotein involved in cell adhesion, is specifically induced in glaucoma TM cells and has been considered the first molecular marker of this ocular disease^35^. In this sense, ELAM-1 mRNA was significantly overexpressed in HTM-3 compared to HTM-5 cells (4.36 ± 0.18-fold; n = 3, p < 0.001; Fig. 1D), further indicating that HTM-3 cells present characteristic changes found in glaucoma. Standard PCR was performed to verify the size of all amplified products and amplification of ELAM-1, MGP and MYOC glaucoma-related genes was checked by sequentiation (data not shown).

### Current density and functional activation of volume-regulated anion channels (VRAC) are altered in glaucomatous HTM-3 cells

**Figure 2:**
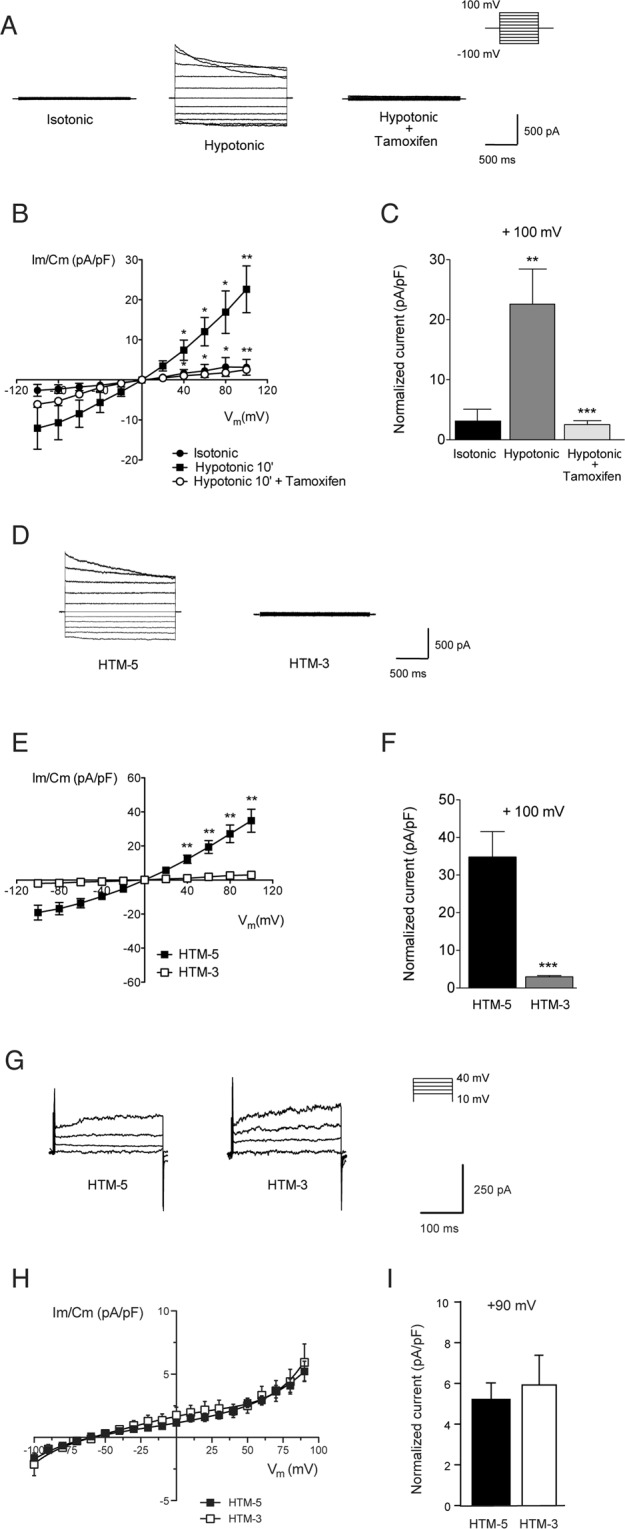
Activation of VRAC and BKCa currents in HTM cell lines. **(A)** Representative VRAC currents recorded in isotonic extracellular medium, and at 10 min in hypotonic extracellular medium (30%) before and after the addition of tamoxifen (100 μM). **(B)** Current-voltage (I-V) curves showing VRAC currents in HTM-5 cells in (⦁) isotonic conditions, (▪) hypotonic conditions, and (◦) hypotonic conditions + tamoxifen. Values are the mean ± SEM of n = 5 independent cells. Current density is determined normalizing currents against cell capacitance (pA/pF). VRAC currents increased significantly after 10 min of hypotonicity (p < 0.001). **p < 0.01, *p < 0.05 isotonic vs. hypotonic, ANOVA *plus* Bonferroni post-tests. Tamoxifen blocked most of VRAC currents activated under hypotonic conditions (p < 0.001). **p < 0.01, *p < 0.05 hypotonic versus hypotonic + tamoxifen, ANOVA *plus* Bonferroni post-tests. **(C)** Peak currents measured at +100 mV in HTM-5 cells at each experimental condition (**p < 0.01 isotonic vs. hypotonic, ***p < 0.001 hypotonic vs. hypotonic + tamoxifen, Student’s t-test). **(D)** Representative experiment showing VRAC currents under hypotonic conditions in control HTM-5 cells and glaucomatous HTM-3 cells (recorded as described in A). **(E)** Normalized I-V relationship activated in (▪) HTM-5 and (▫) HTM-3 at 10 min in hypotonic conditions. Values are mean ± SEM of n = 10 cells (HTM-5) and n = 7 cells (HTM-3). VRAC currents were significantly lower in HTM-3 compared to HTM-5 cells (p < 0.001). **p < 0.01 HTM-3 vs. HTM-5, ANOVA *plus* Bonferroni post-tests. **(F)** Peak currents measured at + 100 mV under hypotonicity (***p < 0.001 HTM-3 vs. HTM-5, Student’s t-test). **(G)** Representative outward K^+^ currents mediated by the high conductance Ca^2+^-activated K^+^ (BKCa) channel in control HTM-5 and glaucomatous HTM-3 cells. **(H)** Current-voltage curves for BKCa in (▪) non-glaucomatous and (▫) glaucomatous HTM cells. Results are the mean ± SEM (n = 11 HTM-5, n = 10 HTM-3). No significant differences were found in current densities between cell lines (Two-way ANOVA and Bonferroni post-tests). **(I)** Peak currents measured at +90 mV in control HTM-5 and glaucomatous HTM-3 cells (glaucoma vs. non-glaucoma, Student’s t-test).

We next studied whether VRAC activity was affected in glaucomatous TM cells compared to the normotensive ones. As shown in Fig. 2A–C, in control HTM-5 cells the current is inactive in isotonic conditions (3.13 ± 1.96 pA/pF at + 100 mV) and rapidly activates after 4 min of hypotonicity, reaching a maximum at 10 min (22.61 ± 5.85 pA/pF) (n = 5, p < 0.001). As expected, VRAC presented a fast activation and then a slow time-dependent inactivation at highly depolarized voltages (Fig. 2A). Current-voltage (I-V) curves exhibited a typical outward rectification and a time-dependent decay (+60 to +100 mV) (Fig. 2B). Moreover, activation of VRAC was reversible when returning to isotonic conditions showing no significant differences to those recorded in isotonic initial medium (n = 5, not shown). When the VRAC blocker tamoxifen was added to hypotonic medium, VRAC currents were significantly reduced to 2.54 ± 0.65 pA/pF (p < 0.0001; Fig. 2C) and outward and inward currents were blocked in a voltage-independent manner along the entire I-V curve (hypotonic vs hypotonic + tamoxifen, p < 0.05, p < 0.01). In HTM-5 cells, VRAC inactivated slowly (τ = 3821.03 ± 262.31 ms) at depolarized voltages (+100 mV) and showed a significant outward rectification (0.52 ± 0.02, n = 10) measured as the ratio between inward/outward currents at −100 mV/+100 mV. In contrast, VRAC activity was significantly diminished in HTM-3 cells: while the current was absent in isotonic conditions (1.50 ± 0.36 pA/pF) (not shown), hypotonic medium only produced a small increase (2.62 ± 0.37 pA/pF; n = 7) compared to the effect found in HTM-5 cells (34.82 ± 6.75 pA/pF; n = 10) at 100 mV (p < 0.0001; Fig. 2D–F). In addition to the reduced current density, HTM-3 cells also showed a delayed activation of VRAC currents compared to those activated in HTM-5 cells at equivalent voltages. To assess whether other ionic conductances were also altered by glaucoma, we recorded total outward K^+^ current in the same cell types. TM cells present a high-conductance Ca^2+^-activated K^+^ (BKCa) channel as previously characterized which contributes to cell volume regulation^36^. All control HMT-5 and glaucomatous HTM-3 cells recorded displayed outward K^+^ currents under isotonic conditions (Fig. 2G,H) and, unlike VRAC, K^+^ currents were not significantly altered between control and glaucomatous HTM cells (HTM-5: 5.22 ± 0.80 pA/pF; HTM-3: 5.93 ± 1.46 pA/pF at + 90 mV, Fig. 2I). These results suggest that glaucomatous cells showed an specific alteration of VRAC, while other currents involved in volume regulation are not equally altered.

### Functional activity of the VRAC is diminished in primary glaucomatous HTM cells

**Figure 3:**
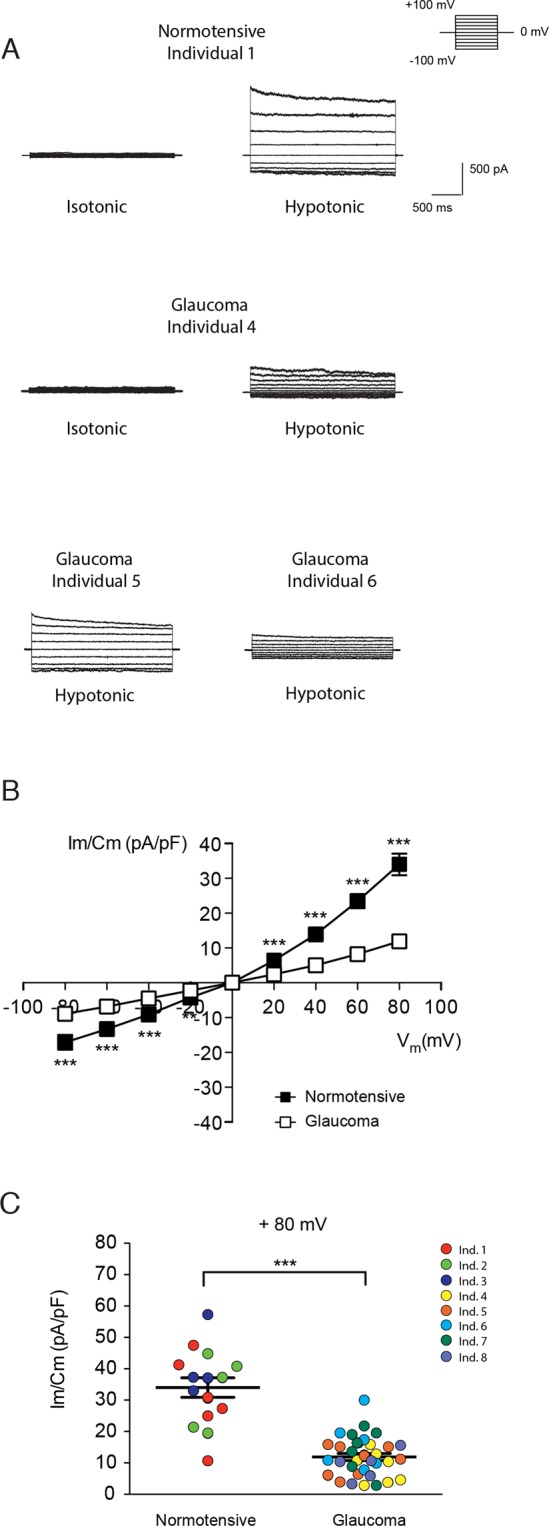
Activation of VRAC currents in primary HTM cells. (**A)** Representative whole-cell currents activated in primary non-glaucomatous (Ind. 1) and glaucomatous HTM cells (Ind. 4, 5 and 6) by depolarizing pulses, from −80 mV to +80 mV in 20 mV steps applied every 5 seconds from a 0 mV holding potential. VRAC currents were recorded in isotonic medium, and after 5 min in hypotonic medium (−30%) (For Ind. 5 and 6 only hypotonic conditions are shown). **(B)** Current-voltage (I-V) curves for VRAC in (▪) non-glaucomatous and (▫) glaucomatous HTM cells under hypotonic conditions for 5 min. Values are shown as the mean ± SEM of non-glaucomatous (n = 15) and glaucomatous (n = 33) primary HTM cells, from three and five individuals, respectively. The hypotonic-mediated increase in VRAC current was significantly smaller in glaucoma compared to control primary HTM cells (p < 0.001). ***p < 0.001 glaucoma vs. non-glaucoma, ANOVA plus Bonferroni post-tests. **(C)** Peak currents measured at +80 mV in control and glaucoma primary HTM cells in hypotonic conditions (***p < 0.001 glaucoma vs. non-glaucoma, Student’s t-test) represented as a dispersion (the colours indicate the eight individuals studied, Table 1).

To further establish a correlation with glaucoma progression and to corroborate the findings obtained in HTM cell lines, we examined the activation of VRAC currents in primary TM cells from five different patients with open-angle glaucoma and three non-glaucomatous human donors. Again, whole-cell currents were recorded in isotonic solution that was replaced by hypotonic medium to activate VRAC (Fig. 3A–C). Under isotonic conditions, non-glaucomatous cells showed small currents of 0.85 ± 0.12 pA/pF at +80 mV (not shown) but after 5 min in hypotonic medium, a significant activation of VRAC was observed (34.02 ± 3.10 pA/pF at +80 mV, Fig. 3B) (p < 0.001 hypotonic vs. isotonic, n = 15). Currents increased further between 5 and 10 min and decreased significantly when returning to isotonic solution (not shown). In glaucomatous cells 33 of 39 exhibited VRAC currents in hypotonic conditions, while no activation was found in the other 6 cells, in contrast to control cells that all showed activation of VRAC. Additionally, VRAC activation was smaller compared to control cells being 1.12 ± 0.14 pA/pF in isotonicity and 11.88 ± 1.10 pA/pF in hypotonicity at +80 mV (Fig. 3B) (p < 0.001 hypotonic vs. isotonic, n = 33). As seen in transformed cell lines, native glaucoma HTM cells showed a delayed activation of VRAC in comparison to control cells at equivalent voltages, in addition to the significant lower current densities. I/V curves showed significant differences between the VRAC current amplitude in open-angle glaucoma and normotensive control cells for the whole positive voltage range (p < 0.001, Fig. 3B). Current density at +80 mV was also represented as a scatter plot for each individual tested (p < 0.001, Fig. 3C).

### Molecular expression of LRRC8A-LRRC8E subunits and the main LRRC8A protein that form VRAC are decreased in glaucomatous HTM- 3 cells

**Figure 4:**
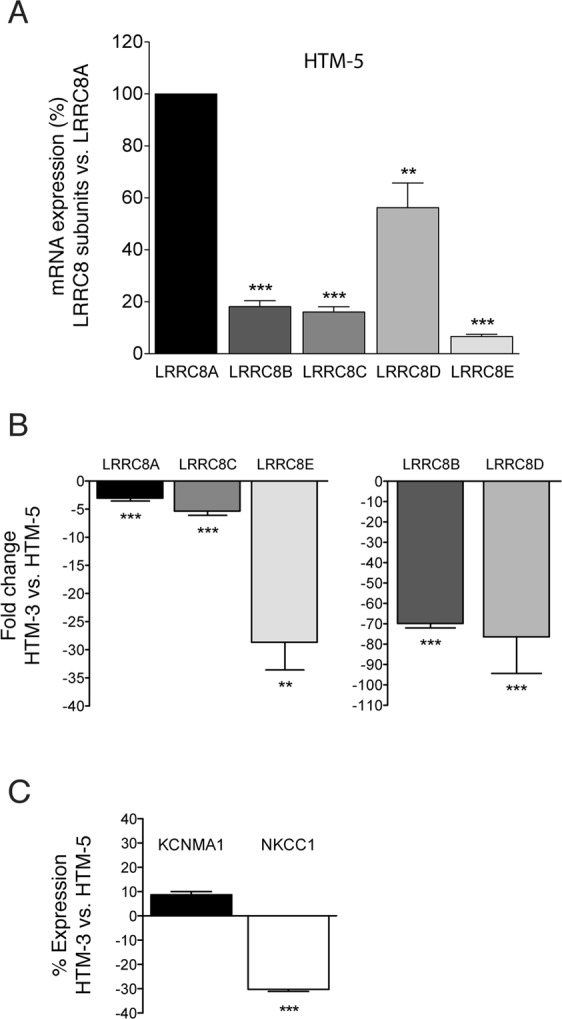
Expression of LRRC8A-LRRC8E, KCNMA1 and NKCC1 genes in HTM cells. **(A)** Relative abundance of LRRC8 mRNAs in HTM-5 cells. Results are shown as the percentage of expression of LRRC8B-LRRC8E compared to the expression of the main subunit LRRC8A normalized against 18S RNA. Values are mean ± SEM of 4 sets of independent experiments performed by triplicate. LRRC8 were expressed in HTM-5, being LRRC8A the most abundant subunit (**p < 0.01, ***p < 0.001, Student’s t-tests). **(B)** LRRC8A-E expression was significantly down-regulated in glaucomatous HTM-3 cells vs. control HTM-5 cells (**p < 0.01, ***p < 0.001, Student’s t-tests). Results are expressed as fold change normalized against 18 S RNA. Values are mean ± SEM of 4 sets of independent experiments done by triplicate. **(C)** Gene expression of KCNMA1 (BKCa) and NKCC1 (Na^+^-K^+^-2Cl^−^) in HTM-3 compared to HTM-5 cells determined by relative quantitative real-time PCR. KCNMA1 expression was not significantly altered in glaucomatous HTM-3 cells (Student’s t-tests) while the expression of NKCC1 was significantly down-regulated in HTM-3 versus HTM-5 (***p < 0.001, Student’s t-tests). Results are expressed as fold change normalized against 18 S RNA. Values are mean ± SEM of n = 3 (KCNMA1) and n = 4 (NKCC1) independent experiments done by triplicate.

To determine whether the decrease in VRAC currents in glaucomatous cells was due to a decreased expression of the mRNA expression of LRRC8 genes, we quantified their relative abundance by qPCR. All LRRC8s were found expressed in HTM-5 cells, being LRRC8A the most expressed one. For comparison purposes, the levels of LRRC8A mRNA were used to normalize the expression of the other subunits. Expression levels of the other subunits, expressed as percentage of LRRC8A (100%) were 18.13 ± 2.29% for LRRC8B, 16.05 ± 2.01% for LRRC8C, 56.24 ± 9.50% for LRRC8D and 6.57 ± 0.85% for LRRC8E (n = 4, Fig. 4A). When the expression profile of LRRC8A-LRRC8E was determined in glaucomatous HTM-3 cells, a significant decrease in all subunits was found compared to HTM-5 cells (in fold change): −3.02 ± 0.52 in LRRC8A, −69.83 ± 1.54 in LRRC8B, −5.34 ± 1.04 in LRRC8C, -76.42 ± 12.68 in LRRC8D and −28.67 ± 3.48 in LRRC8E (n = 4, Fig. 4B). Standard PCR was done to verify the size of amplified products (not shown). We also determined the mRNA expression of the BKCa channel and the Na^+^-K^+^-2Cl^−^ cotransporter, two of the main proteins involved in the TM cell volume regulation together with VRAC. According to the channel activity, the expression of KCNMA1 (coding for the alpha-1 subunit of the BKCa) was not significantly altered in glaucomatous HTM-3 cells (8.7 ± 1.3% from the expression in HTM-5). In contrast, NKCC1 (coding for the Na^+^-K^+^-2Cl^−^) was significantly down-regulated in HTM-3 cells (−30.2 ± 0.9% from HTM-5, Fig. 4C), similarly to what has been previously reported^37^.

**Figure 5:**
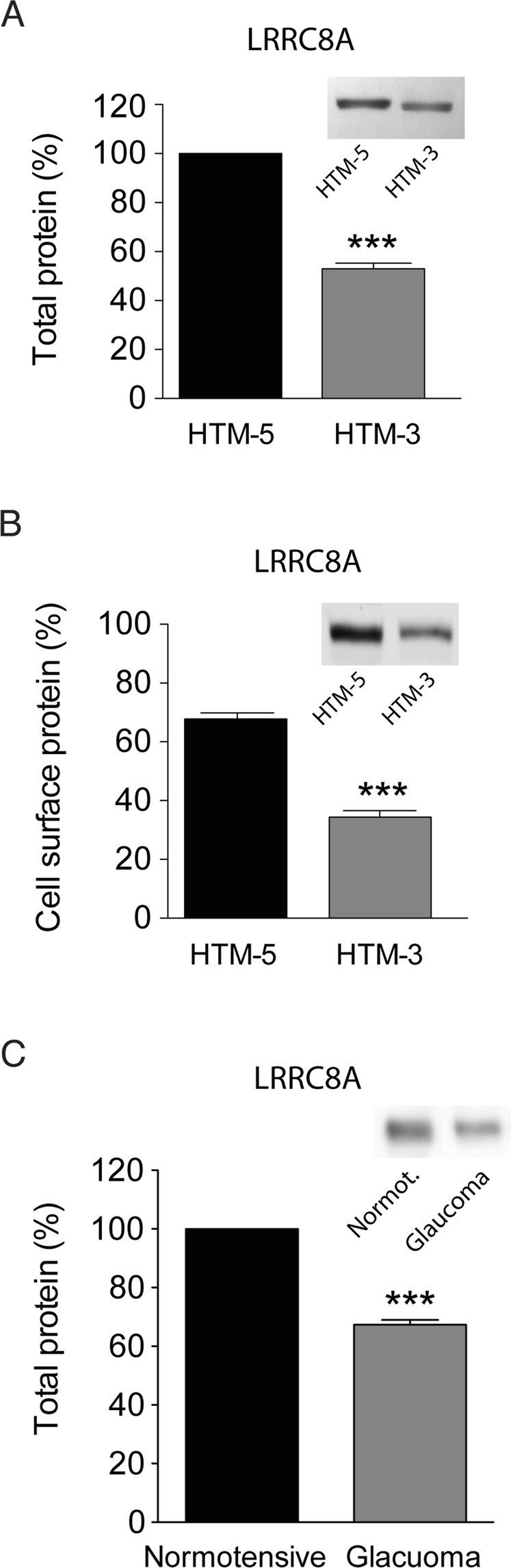
Quantification of LRRC8A protein at HTM cells. **(A)** LRRC8A was significantly lower in HTM-3 compared to HTM-5. Data are expressed as the percentage of LRRC8A in HTM-3 normalized to its amount in HTM-5. Values are mean ± SEM of 7 protein extractions (***p < 0.001, Student’s t-tests). Total protein was previously quantified to load the same amount of protein of the two cell lines and the LRRC8A signal was normalized by β-actin. *Inset*: Representative western blot showing LRRC8A (95 KDa) in HTM-5 and HTM-3 cell extracts. Full-length blot is presented in Supplementary Fig. 5A. **(B)** Relative abundance of LRRC8A protein at cell surface in relation to total LRRC8A protein expressed as a percentage. Quantification shows a lower amount of LRRC8A protein at the PM of HTM-3 cells compared to that at HTM-5 cells. Values are mean ± SEM of 5 independent experiments (***p < 0.001, Student’s t-tests). *Inset*: Representative WB of cell surface biotinylation showing LRRC8A. Full-length blot is shown in Suppl. Figure 5B. A set of experiments without biotin was done as a negative control (not shown). **(C)** LRRC8A protein was also significantly lower in primary TM cells from glaucoma patients in comparison to non-glaucomatous donors. Protein extracts were previously quantified to load the same amount of each cell sample from Ind. 1–3 (control, normotensive) and Ind. 5–8 (glaucoma), and LRRC8A signal normalized by β-actin. Values are mean ± SEM of two different film intensities of two western blots from the same protein extracts (***p < 0.001, Student’s t-tests). *Inset*: Representative WB showing LRRC8A in control (Ind. 1) and glaucoma (Ind. 5) protein samples. Full-length blots are in Supplementary Fig. 5C.

At the protein level, LRRC8A was also decreased by 52.91 ± 2.34% in HTM-3 compared to HTM-5 cells (n = 7 protein extractions, p < 0.001; Fig. 5A). Despite the decreased level of LRRC8A mRNA and total protein, it could still be possible that the amount of protein reaching the PM would be sufficient to produce large VRAC currents similar to those activated in normotensive cells. Hence, we quantified the protein levels of LRRC8A at the PM by biotinylation of cell-surface proteins. These studies revealed that while the proportion of LRRC8A on the PM in HTM-5 cells was 67.83 ± 2.08% of the total protein, this proportion falls to 34.36 ± 2.25% in HTM-3 cells (n = 5 independent biotinylation experiments, p < 0.001; Fig. 5B). Interestingly, LRRC8A protein was significantly diminished by 32.65 ± 1.61% in primary HTM cells derived from patients with open-angle glaucoma (Ind. 5–8) compared to native HTM cells from normotensive donors (Ind. 1–3) (p < 0.001; Fig. 5C). Altogether our data suggest that the decreased VRAC currents in glaucomatous cells is due to a decrease in LRRC8 mRNA expression combined with a reduction in the total and surface LRRC8A protein.

### Cell volume regulation is impaired in glaucomatous HTM-3 cells

**Figure 6:**
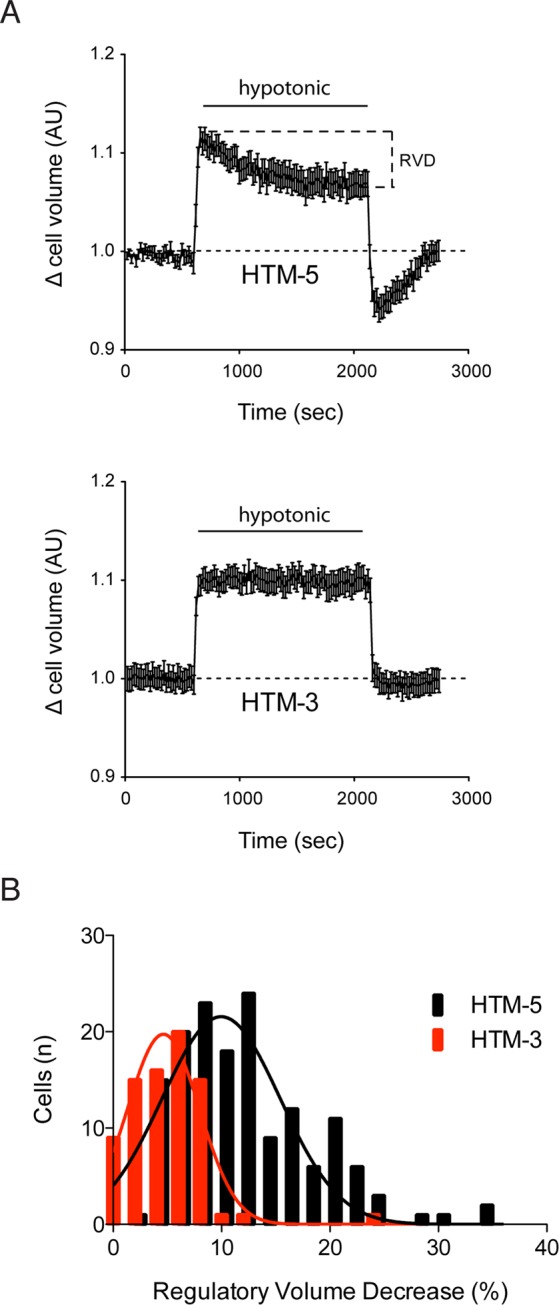
Volume regulation of HTM cells. **(A)** RVD activated in HTM-5 and HTM-3 cells was measured using calcein as an index of cellular volume (see Material and Methods for details). After baseline at isotonic conditions (10 min), swollen was induced by application of hypotonic bath solution (27% for 30 min) to activate the RVD. Glaucomatous HTM-3 cells showed an inappreciable RVD in comparison to the RVD activated in control HTM-5 cells. Results are plotted as mean ± SEM of a set of two representative experiments (n = 17 HTM-5 cells, n = 15 HTM-3 cells). **(B)** RVD is determined as the percentage of cell volume recovery during the hypotonic period being significantly lower in HTM-3 cells (n = 79) than in HTM-5 cells (n = 153). Values are represented versus the distribution of the number of cells in each group (***p < 0.001, Student’s t-tests).

We measured the Regulatory Volume Decrease (RVD) activated in HTM cells upon hypotonic shock by means of calcein fluorescence. After 5 min in isotonic medium to establish the initial baseline, HTM cells were exposed to hypotonic medium (27%) to swell the cells and trigger a subsequent RVD. As predicted by the low VRAC currents, the RVD was significantly impaired in glaucomatous cells compared to that activated in control cells (Fig. 6A). Hence, HTM-5 exhibited an RVD of 11.91 ± 0.50% (range 2.15–34.02%) after 30 min of hypotonicity (n = 152 cells) while in HTM-3 this value was of 4.99 ± 0.40% (range 0.04–24.19%; n = 79 cells; Fig. 6B). When isotonic solution was restored, HTM-5 volume decreased under baseline and slowly returned to initial resting values. This rebound effect was proportional to the activated RVD (Fig. 6A). Alteration of the regulatory mechanisms to restore TM cell volume in glaucoma may compromise the physiological maintenance of the AH outflow.

### Down-regulation of LRRC8 genes in human anterior ocular segments perfused under elevated pressure conditions

**Figure 7:**
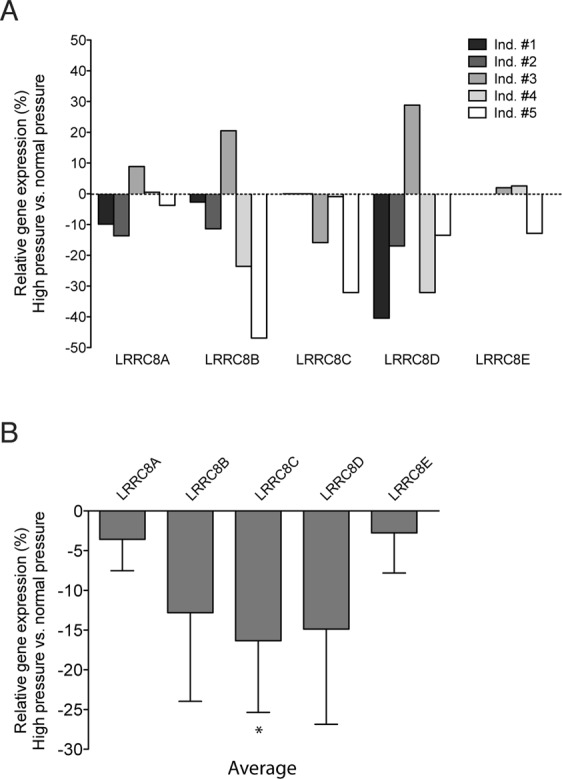
Differential expression of LRRC8A-LRRC8E genes under pressure in human TM. Paired ocular anterior segments from five donors were perfused at high P or physiological P during 7 d as in Material and Methods. For each individual, the relative expression of LRRC8 genes in human TM tissues was determined with Affymetrix GeneChips U95Av2 (individuals #1 and #2) or U133Plus2.0 (Ind. #3, #4, and #5). **(A)** Bars represent changes in the LRRC8 expression by high P treatment in each individual, plotted separately to assess individual molecular response to pressure. Because the two types of microarrays contain ≈12,625 and 54,678 probes respectively, LRR8C and LRRC8E were not detected in the U95Av2 platform (Ind. #1 and #2). **(B)** Expression of LRRC8 genes as mean ± SEM shows their tendency to decrease by elevated pressure insults. LRRC8C down-regulation is the only statistically significant because of the differential molecular response to pressure of the individual #3. (*p = 0.038 by the Student’s t-test).

To corroborate the relevance of our results from cellular models in the pathophysiology of glaucoma, we took advantage of previously performed microarray experiments in human TM tissue from ocular anterior segments perfused at normal/elevated pressure for 7 days. We reanalysed the microarray data looking for changes in the newly identified VRAC molecular components^31^. Analysis of DNA microarrays showed a significant down-regulation of LRRC8B, LRRC8C and LRRC8D genes in response to high pressure in four out of the five individuals tested. These results further reinforce the finding that ocular hypertension impairs VRAC expression and function in the TM tissue (Fig. 7).

### The expression of LRRC8 genes and the LRRC8A protein is induced in a glaucoma model *in vitro*

It is well known that corticosteroids cause a specific form of glaucoma (steroid-induced open angle glaucoma)^38^, characterized by expression of specific genes (e.g. Miocylin), extracellular matrix (ECM) remodelling and an important up-regulation of a large number of genes. To assess if VRAC was differentially affected in this type of glaucoma, we used a cellular model by incubating HTM-5 cells with 100 nM DEX (or vehicle) during 3 or 7 days. Real-time qPCRs showed that the five LRRC8A-E isoforms of VRAC were mostly induced by DEX. Fold changes were 1.88 ± 0.74 in LRRC8A, 3.35 ± 1.11 in LRRC8B (p < 0.05), 1.55 ± 0.50 in LRRC8C, 2.44 ± 0.24 in LRRC8D (p < 0.001) and 3.62 ± 1.97 in LRRC8E at 3 days of DEX and 2.68 ± 1.16 in LRRC8A (p < 0.05), 2.21 ± 0.19 in LRRC8B (p < 0.001), 1.15 ± 0.22 in LRRC8C, 5.76 ± 2.21 in LRRC8D (p < 0.05) and 2.39 ± 0.70 in LRRC8E (p < 0.05) after 7 days of DEX (n = 3–5 experiments by triplicate, Student’s t-tests; data not shown). Consequently, western blot of protein extracts from vehicle and DEX-treated HTM-5 cells were performed with LRRC8A and β-actin antibodies. After normalization, levels of LRRC8A protein were found induced by 33.38% (p = 0.167) at 3 days and 106.17% (p = 0.014) at 7 days of DEX compared to vehicle. Thus, LRRC8 mRNA and the LRRC8A protein were altered by DEX as it occurs in glaucomatous cells, but in the opposite direction. Because MYOC is induced by DEX specifically in the TM, its expression was used to validate the effect of DEX on HTM-5 cells. As expected, MYOC was induced by 4.65 ± 1.22-fold (p < 0.05) and 8.26 ± 1.63-fold (p < 0.01) at 3 and 7 days of DEX respectively, similarly to previously reported data^39^.

## Discussion

Volume-regulated anion channels are fundamental to maintain cellular volume in vertebrate cells^40^. We and others, previously demonstrated the participation of this channel in volume regulation of TM cells and aqueous outflow^5,6^ but it was not until recently that LRRC8 proteins have been identified as the molecular components of VRAC^15,16^. LRRC8A is the most important subunit and required with at least one of the other LRRC8A-LRRC8E subunits to form functional VRAC^15,16,25,41^. This finding permitted us to characterize its expression and activity in the pathophysiology of glaucoma. Using established TM cell lines, allowed us to have enough starting material to perform different electrophysiological, biochemical, and imaging techniques. Because cell lines might undergo some changes due to the culture conditions, we validated them by quantifying the expression of different well-known markers present in glaucomatous TM cells. MYOC was discovered when its expression was induced by DEX in human TM. Importantly, DEX treatment causes ocular hypertension and today it is accepted that DEX-induced overexpression of MYOC is associated to steroid-induced glaucoma^39^. As expected, a chronic treatment on normotensive HTM-5 cells with DEX produced the induction of MYOC mRNA and protein levels, which is specific of TM cells^42^. Then, we rationally selected MGP and ELAM-1 genes, known to be relevant to outflow function, in order to validate HTM-3 cells. MGP gene encodes an inhibitor of calcification that protects soft tissues like vascular smooth muscle cells from mineralization^43^. MGP is highly abundant in adult TM but its expression decreases in glaucomatous TM cells^30^, as we corroborated in HTM-3 cells. Also, the first molecular marker for glaucoma, ELAM-1^35^, was found induced in HTM-3 glaucomatous cells compared to control cells, where it was almost absent. Altogether, our data show that HTM cells present characteristic features found in normal TM and several of the changes that occur during POAG.

VRAC was first recorded in human T lymphocytes^44^ and since then, biophysical properties have been characterized in several cell types^8^. Similarly to previous data in primary bovine trabecular meshwork cells (BTM)^6,12^ and HTM cells^5^, the HTM-5 cell line present currents with characteristic electrophysiological properties of VRAC (outward rectification, voltage-dependent inactivation, and inhibition by tamoxifen). To date, mechanisms by which cell swelling activates VRAC are far from being understood. The debate is whether the channel itself senses the change in volume or whether changes in membrane tension or other factors are required for gating^8^. A decreased ionic strength^45^ and other processes independent to cellular volume like shear stress^46^, intracellular GTPγS^47^, purinergic signalling^48^ and ROS^49^ also activate VRAC. Now, it is known that specific biophysical properties of VRAC depend on the type of cell, mainly due to the diversity generated by the combination of LRRC8 subunits, and that distinct LRRC8 proteins can coexist in a cell to form heterohexameric VRAC with specific intrinsic properties^16,22,25,41^. Currents with stronger outward rectification have been recorded in HEK cells lacking LRRC8B, LRRC8C and LRRC8E in comparison to HEK cells without LRRC8B, LRRC8D, LRRC8E and LRRC8B LRRC8C LRRC8D. Moreover, and in contrast to LRRC8C-mediated currents, LRRC8D- and LRRC8E subunits generate currents that inactivate faster and at less positive potentials^16^. In control HTM-5 cells, VRAC currents show a significant outward rectification and a slow inactivation compared to previously published results^16,22^. All the LRRC8A-LRRC8E mRNAs have been detected in control HTM-5 cells, where the obligatory subunit for VRAC, LRRC8A, is highly expressed. However, the particular stoichiometry of the VRAC complexes assembled in these cells would require a complete and detailed biophysical characterization.

In glaucomatous HTM-3 cells VRAC currents show a much lower density and a delayed activation compared to those activated in control cells. Notably, the activity of VRAC was also decreased in native TM cells from five patients with open-angle glaucoma (Ind. 4–8). Individual 4 was a case of secondary glaucoma due to idiopathic uveitis (IOP 35 mmHg pre-trabeculectomy and 14 mmHg at 3 months after surgery without pharmacological treatment). This patient suffered several outbreaks of uveitis in the past for which she received treatment with cortisone. It is not possible to discern whether ocular hypertension was caused by inflammation or corticosteroids. Because the maximum pharmacological treatment did not allow good control of the hypertension, surgery was necessary. At the time of trabeculectomy, she did not receive steroids for ten years. It is important to note that the other glaucomatous native HTM cell lines are derived from POAG patients (Ind. 5–8). All of them were treated with beta-blockers and carbonic anhydrase inhibitors, plus prostaglandins (Ind. 5–7) before surgery. In contrast to VRAC, outward K^+^ currents mediated by BKCa were unaffected in HTM-3 cells. This indicates that the down-regulation of VRAC function in HTM-3 is specific and not a general effect on other proteins/channels. We previously found that RVD is markedly attenuated by tamoxifen (a VRAC blocker) in BTM cells^12^. Accordingly, inhibition of Cl^−^ or K^+^ channels with NPPB or TEA respectively, is sufficient to abolish RVD in HTM cells, while it is only partially reduced blocking the K^+^-Cl^−^-symport with DIOA^5^. Therefore, although the amount of LRRC8A protein could be acceptable, the decreased activity of VRAC may largely impair the regulation of cell volume. The expression of Na^+^-K^+^-2Cl^−^, one of the main transporters of the RVD, is also decreased in HTM-3 cells by 29%, as described in TM cells from glaucoma patients^37^. These proteins might contribute to the decreased ability to regulate the volume of glaucomatous cells. Interestingly, the same authors that found the Na^+^-K^+^-2Cl^−^ diminished in glaucoma TM cells, described that its expression and activity was stimulated in HTM and BTM cells after DEX exposure^50^. This proves once again that some genes altered in glaucoma are differentially affected by DEX, which induces its expression.

Approximately 40% of patients who are long-term treated with corticosteroids develop a pronounced rise of IOP and POAG and up to 90% of POAG cases are considered “steroid responders”^51^. Reorganization of the cytoskeleton^52^, an increased ECM deposition^53^, and altered synthesis of specific proteins^54^ in TM cells are some of the mechanisms proposed for steroid-induced glaucoma. Expression of MYOC have been found induced in TM in almost half of the POAG cases^55^ and MYOC protein is enhanced in the TM by the steroid DEX^39^. For all this, the use of DEX has been used as a glaucoma model *in vitro*. In our study, DEX induced MYOC expression in HTM-5 cells, showing a consistent response of this control cell line to steroids. We also found an enhanced expression of LRRC8A-E genes, a response in the opposite direction to the found in cells from POAG. There is much evidence that gene expression is changed in TM cells in response to DEX^42,56,57^. Borrás and coworkers reported expression changes in human TM tissues in response to DEX for 6–8 days by microarray analysis. Among the genes with expression levels than changed more than 20-fold by DEX, a higher number were up-regulated (40) than down-regulated (18). In addition, a significant set of the genes induced by DEX were located in chromosomal regions linked to glaucoma^42^. Rozsa and collaborators also identified a larger number of genes enhanced than decreased after 21 days of DEX treatment in the TM. In fact, exposure of HTM to steroids for weeks increases the synthesis of several proteins that may be relevant for trabecular funcion^39,54,58^. Thus, corticoids tend to increase above all, the expression of specific proteins that could be associated to the development of steroid-induced glaucoma. Because a normal cytoskeleton is needed for the RVD mechanism^59^, we used Phalloidin 555 to study F-actin on glaucomatous and control TM cell lines but we did not find evident changes (not shown). However, we can not discard that other mechanical properties such as stiffness of the TM, that have been shown to differ in POAG, could compromise the correct regulation of cell volume^60^.

Grant and co-workers previously showed that VRAC currents were activated in hypotonic conditions but they did not found significant differences in VRAC between normotensive (NTM) and glaucomatous (GTM) TM cells, although a tendency might exist^61^. Surprisingly, they report VRAC activation in isotonic conditions, especially in NTM cells. In none of our studies with primary HTM, BTM or transformed TM cells VRAC was active at rest (isotonic) as it has been widely published^62^. These differences could be due to technical reasons and the use of non-selective chloride solutions. Solutions containing with NMDG-Cl^−^ (as used here) are a standard when recording this type of currents. Also, biophysical and pharmacological properties indicate that our chloride currents are mediated by VRAC^6,12^. Our data indicate that the reduced VRAC currents in glaucomatous cells are the consequence of a combined decrease in mRNA of all LRRC8 subunits and LRRC8A protein. Importantly, microarray data from ref.^31^. revealed a slight but significant down-regulation of LRRC8B, LRRC8D and LRRC8E genes in TM tissues in response to pressure insults. Therefore, the expression pattern of LRRC8 under high-pressure conditions changed in the same direction than in glaucoma but in a lesser extent, regardless some individual differences. An altered composition of AH and/or an increased shear stress caused the aqueous outflow under pressure could contribute to the differential gene expression found in glaucoma.

The subunit composition of the functional complexes dictates the substrate selectivity, since LRRC8A/LRRC8D expression is associated to the release of taurine and LRRC8A, LRRC8C and LRRC8E heteromers favour anion permeation^41^. LRRC8D, but not LRRC8C or LRRC8E, is also required for the blasticidin uptake^26^. Therefore, the called volume-sensitive organic osmolyte-anion channel (VSOAC) and VRAC would refer to differently composed LRRC8 heteromers^41^. Because *LRRC8D*^−/−^ HEK cells show diminished swelling-induced taurine efflux and a decreased RVD but normal anion currents, organic metabolites could participate in volume regulation^16^. In addition, a lower amount of LRRC8A has been associated to a lower capacity to modulate cell volume^15–17^. In this regard, we have found a significant expression of LRRC8A and LRRC8D mRNAs in HTM-5 cells that show the capacity to restore part of cell volume in hypotonic conditions. Otherwise, Kunzelmann and collaborators probed that LRRC8A had little impact on volume regulation of HeLa cells. LRRC8A siRNAs and the channel inhibitor NS3728 slightly attenuate cell volume regulation but it was strongly delayed by the inhibition of the K^+^-Cl^−^ symport. In a similar way, RVD was detected in HCT116 cells lacking LRRC8A but not in the BHY cell line without this subunit^63^. Therefore, participation of LRRC8A to cell volume regulation may depend on the cell type. Noteworthy, maximal VRAC currents were observed at 2.5 min of hypotonic medium (33%) and cell volume recovery was completed at 3.5 min in HeLa^63^. The temperature at which the experiments were done (37 °C) could explain fast VRAC currents and the strong RVD since it is confirmed that both are attenuated at 20 °C. In this sense, BTM cells exhibit a RVD of 21% after 30 min of hypotonicity (40%) at 22–23 °C^6^ consistent to that measured in human TM cells^5^. Similarly, we recorded maximal VRAC currents at 15 min and a RVD of 11% after 30 min of 27% hypotonicity in HTM-5 cells. Although the molecular basis of cell volume regulation remains unknown the role of the temperature-sensitive PLA_2_ has been suggested to be important for current density and time-dependent inactivation of VRAC and for RVD^63^. Thus, VRAC is a key player of RVD activated in trabecular cells, that also implies activation of the BKCa, the K^+^-Cl^−^ symport, the Na^+^-K^+^-Cl^−^ cotransporter, and the Na^+^/H^+^ antiport^5,6^. Taken all the data into account, VRAC is probably essential for volume regulation in many cell types including TM cells^15,16,40,41^ but not quite indispensable in others^63^. Because BKCa function is not altered in HTM-3 cells, we propose that at least VRAC is required for TM cell volume regulation.

Volume of trabecular cells contribute to the aqueous outflow facility^4–6,10,11^. As we determined previously, inhibition of VRAC and BKCa reduced the ability to recover outflow facility under hypotonic conditions while specific activation of BKCa showed the opposite effect^6^. Consequently, down-regulation of LRRC8 subunits in glaucomatous TM cells could explain the impaired modulation of cellular volume underlying an increased resistance to aqueous outflow. Aside from the volume regulation, LRRC8-mediated channels are associated to processes like transport of modulators, extracellular signal transduction and cell proliferation^40^. The cellularity of the TM tissue decreases with age^64^ and decreases further in eyes from POAG patients^65^. In fact, laser trabeculoplasty stimulates the replication of TM cells as a part of its mechanism of action^66^. A reduced number of functional VRAC in glaucomatous trabecular cells could be associated to a lower degree of proliferation.

In conclusion, glaucomatous TM cells shows a decreased VRAC activity due to a down-regulation of LRRC8 mRNA and protein, which impairs cell volume regulation. This might be associated to the altered AH outflow in open angle glaucoma patients. Finding activators of LRRC8-channels could be very useful in the trabecular physiology as they could be used to manage ocular hypertension and glaucoma.

## Supplementary information

Supplementary Information

## References

[1] Quigley HA (2011). Glaucoma. Lancet.

[2] Lütjen-Drecoll E (2000). Importance of trabecular meshwork changes in the pathogenesis of primary open-angle glaucoma. J Glaucoma.

[3] Wiederholt M, Thieme H, Stumpff F (2000). The regulation of trabecular meshwork and ciliary muscle contractility. Prog. Retin. Eye Res..

[4] Gual A (1997). Effects of time of storage, albumin, and osmolality changes on outflow facility (C) of bovine anterior segment *in vitro*. Investig. Ophthalmol. Vis. Sci..

[5] Mitchell CH, Fleischhauer JC, Stamer WD, Peterson-Yantorno K, Civan MM (2002). Human trabecular meshwork cell volume regulation. Am. J. Physiol. Cell Physiol..

[6] Soto D (2004). Modulation of aqueous humor outflow by ionic mechanisms involved in trabecular meshwork cell volume regulation. Invest. Ophthalmol. Vis. Sci..

[7] Saccà SC, Pulliero A, Izzotti A (2015). The Dysfunction of the Trabecular Meshwork During Glaucoma Course. J. Cell. Physiol..

[8] Hoffmann EK, Lambert IH, Pedersen SF (2009). Physiology of Cell Volume Regulation in Vertebrates. Physiol Rev.

[9] Okada Y (2001). Receptor-mediated control of regulatory volume decrease (RVD) and apoptotic volume decrease (AVD). J. Physiol..

[10] O’Donnell ME, Brandt JD, Curry FR (1995). Na-K-Cl cotransport regulates intracellular volume and monolayer permeability of trabecular meshwork cells. Am. J. Physiol..

[11] Al-Aswad LA (1999). Effects of Na-K-2Cl cotransport regulators on outflow facility in calf and human eyes *in vitro*. *Investig*. *Ophthalmol*. Vis. Sci..

[12] Comes N (2006). Identification and functional characterization of ClC-2 chloride channels in trabecular meshwork cells. Exp. Eye Res..

[13] Nilius B (1994). Volume-activated Cl- currents in different mammalian non-excitable cell types. Pflugers Arch.

[14] Pedersen SF, Klausen TK, Nilius B (2015). The identification of a volume-regulated anion channel: An amazing Odyssey. Acta Physiol..

[15] Qiu Z (2014). SWELL1, a plasma membrane protein, is an essential component of volume-regulated anion channel. Cell.

[16] Voss FK (2014). Identification of LRRC8 heteromers as an essential component of the volume-regulated anion channel VRAC. Science.

[17] Hyzinski-García MC, Rudkouskaya A, Mongin AA (2014). LRRC8A protein is indispensable for swelling-activated and ATP-induced release of excitatory amino acids in rat astrocytes. J Physiol.

[18] Sawada A (2003). A congenital mutation of the novel gene LRRC8 causes agammaglobulinemia in humans. J. Clin. Invest..

[19] Smits G, Kajava AV (2004). LRRC8 extracellular domain is composed of 17 leucine-rich repeats. Mol. Immunol..

[20] Kubota K (2004). LRRC8 involved in B cell development belongs to a novel family of leucine-rich repeat proteins. FEBS Lett..

[21] Abascal F, Zardoya R (2012). LRRC8 proteins share a common ancestor with pannexins, and may form hexameric channels involved in cell-cell communication. BioEssays.

[22] Syeda R (2016). LRRC8 Proteins Form Volume-Regulated Anion Channels that Sense Ionic Strength. Cell.

[23] Gaitán-Peñas H (2016). Investigation of LRRC8-Mediated Volume-Regulated Anion Currents in Xenopus Oocytes. Biophys. J..

[24] Gaitán-Peñas, H., Pusch, M. & Estévez, R. Expression of LRRC8/VRAC currents in Xenopus oocytes: Advantages and caveats. *Int*. *J*. *Mol*. *Sci*. **19** (2018). 10.3390/ijms19030719 PMC5877580 29498698

[25] Lutter D, Ullrich F, Lueck JC, Kempa S, Jentsch TJ (2017). Selective transport of neurotransmitters and –modulators by distinct volume-regulated LRRC8 anion channels. J Cell Sci..

[26] Lee CC, Freinkman E, Sabatini DM, Ploegh HL (2014). The protein synthesis inhibitor blasticidin s enters mammalian cells via Leucine-rich repeat-containing protein 8D. J. Biol. Chem..

[27] Pang I, Shade D, Clark A, Steely H, DeSantis L (1994). Preliminary characterization of a transformed cell strain derived from human trabecular meshwork. Curr Eye Res..

[28] Stamer DW, Seftor RE, Williams SK, Samaha HA, Snyder RW (1995). Isolation and culture of human trabecular meshwork cells by extracellular matrix digestion. Curr Eye Res..

[29] Stamer DW, Roberts BC, Epstein DL, Allingham RR (2000). Isolation of primary open-angle glaucomatous trabecular meshwork cells from whole eye tissue. Curr Eye Res..

[30] Xue W, Comes N, Borrás T (2007). Presence of an established calcification marker in trabecular meshwork tissue of glaucoma donors. Investig. Ophthalmol. Vis. Sci..

[31] Comes N, Borrás T (2010). Individual molecular response to elevated intraocular pressure in perfused postmortem human eyes. Physiol Genomics.

[32] Crowe WE, Altamirano J, Huerto L, Alvarez-Leefmans FJ (1995). Volume changes in single N1E-115 neuroblastoma cells measured with a fluorescent probe. Neuroscience.

[33] Polansky, J. R., Kurtz, R. M., Fauss, D. J., Kim, R. Y. & Bloom, E. In *Glaucoma Update IV* (ed. Krieglstein, G. K.) 20–29 (Springer-Verlag 1991).

[34] Borrás T, Comes N (2009). Evidence for a calcification process in the trabecular meshwork. Experimental Eye Research.

[35] Wang N, Chintala SK, Fini ME, Schuman JS (2001). Activation of a tissue-specific stress response in the aqueous outflow pathway of the eye defines the glaucoma disease phenotype. Nat Med.

[36] Gasull X (2003). Cell membrane stretch modulates the high-conductance Ca_2_+-activated K+ channel in bovine trabecular meshwork cells. Invest. Ophthalmol. Vis. Sci..

[37] Putney LK, Brandt JD, O’Donnell ME (1999). Na-K-Cl cotransport in normal and glaucomatous human trabecular meshwork cells. Investig. Ophthalmol. Vis. Sci..

[38] Weinreb RN, Polansky JR, Kramer SG, Baxter JD (1985). Acute effects of dexamethasone on intraocular pressure in glaucoma. Invest. Ophthalmol. Vis. Sci..

[39] Nguyen TD (1998). Gene structure and properties of TIGR, an olfactomedin-related glycoprotein cloned from glucocorticoid-induced trabecular meshwork cells. J. Biol. Chem..

[40] Jentsch TJ, Lutter D, Planells-Cases R, Ullrich F, Voss FK (2016). VRAC: molecular identification as LRRC8 heteromers with differential functions. Pflugers Arch. Eur. J. Physiol..

[41] Planells-Cases R (2015). Subunit composition of VRAC channels determines substrate specificity and cellular resistance to Pt-based anti-cancer drugs. EMBO J..

[42] Lo WR (2003). Tissue differential microarray analysis of dexamethasone induction reveals potential mechanisms of steroid glaucoma. Invest. Ophthalmol. Vis. Sci..

[43] Proudfoot D, Shanahan CM (2006). Molecular mechanisms mediating vascular calcification: role of matrix Gla protein. Nephrology (Carlton)..

[44] Cahalan MD, Lewis RS (1988). Role of potassium and chloride channels in volume regulation by T lymphocytes. Soc. Gen. Physiol. Ser..

[45] Syeda, R. *et al*. LRRC8 Proteins Form Volume-Regulated Anion Channels that Sense Ionic Strength Article LRRC8 Proteins Form Volume-Regulated Anion Channels that Sense Ionic Strength. *Cell* 499–511 10.1016/j.cell.2015.12.031 (2016). 10.1016/j.cell.2015.12.031 PMC4733249 26824658

[46] Nakao M, Ono K, Fujisawa S, Iijima T (1999). Mechanical stress-induced Ca^2+^ entry and Cl^−^ current in cultured human aortic endothelial cells. Am. J. Physiol..

[47] Voets T (1998). Regulation of a swelling-activated chloride current in bovine endothelium by protein tyrosine phosphorylation and G proteins. J. Physiol..

[48] Wang YU, Roman R, Lidofskyt SD, Fitz JG (1996). Autocrine signaling through ATP release represents a novel mechanism for cell volume regulation. Proc. Natl. Acad. Sci..

[49] Shimizu T, Numata T, Okada Y (2004). A role of reactive oxygen species in apoptotic activation of volume-sensitive Cl- channel. Proc. Natl. Acad. Sci..

[50] Putney LK, Brandt JD, O’Donnell ME (1997). Effects of dexamethasone on sodium-potassium-chloride cotransport in trabecular meshwork cells. Investig. Opthalmology Vis. Sci..

[51] Johnson, D. H. *Corticosteroid glaucoma*. *Chandler and Grant’s Glaucoma*. (1997).

[52] Wilson K, McCartney MD, Miggans ST, Clark AF (1993). Dexamethasone induced ultrastructural changes in cultured human trabecular meshwork cells. Curr Eye Res..

[53] Zhou L, Li Y, Yue BYJT (1998). Glucocorticoid effects on extracellular matrix proteins and integrins in bovine trabecular meshwork cells in relation to glaucoma. Int. J. Mol. Med..

[54] Partridge CA, Weinstein BI, Southren AL, Gerritsen ME (1989). Dexamethasone induces specific proteins in human trabecular meshwork cells. Invest. Ophthalmol. Vis. Sci..

[55] Shimizu. S (2000). Age-dependent prevalence of mutations at the GLC1A locus in primary open-angle glaucoma. Am. J. Ophthalmol..

[56] Ishibashi T (2002). cDNA microarray analysis of gene expression changes induced by dexamethasone in cultured human trabecular meshwork cells. Invest Ophthalmol.Vis.Sci.

[57] Rozsa FW (2006). Gene expression profile of human trabecular meshwork cells in response to long-term dexamethasone exposure. Mol. Vis..

[58] Polansky, J. R., Bloom, E., Knoami, D., Weinreb, R. W. & Alvarado, J. A. *Cultured human trabecular cells: evaluation of hormonal and pharmacological responses in vitro*. *In Recent Advances In Glaucoma*. (1984).

[59] Pedersen SF, Mills JW, Hoffmann EK (1999). Role of the F-actin cytoskeleton in the RVD and RVI processes in Ehrlich ascites tumor cells. Exp. Cell Res..

[60] Wang K (2018). The relationship between outflow resistance and trabecular meshwork stiffness in mice. Sci. Rep..

[61] Grant J, Tran V, Bhattacharya SK, Bianchi L (2013). Ionic Currents of Human Trabecular Meshwork Cells from Control and Glaucoma Subjects. J. Membr. Biol..

[62] Bond TD, Ambikapathy S, Mohammad S, Valverde MA (1998). Osmosensitive Cl- currents and their relevance to regulatory volume decrease in human intestinal T84 cells: outwardly vs. inwardly rectifying currents. J. Physiol..

[63] Sirianant L (2016). Non-essential contribution of LRRC8A to volume regulation. Pflugers Arch. Eur. J. Physiol..

[64] Grierson I, Howes RC (1987). Age-related depletion of the cell population in the human trabecular meshwork. Eye.

[65] Alvarado J, Murphy C, Juster R (1984). Trabecular meshwork cellularity in primary open-angle glaucoma and nonglaucomatous normals. Ophthalmology.

[66] Acott T (1989). Trabecular repopulation by anterior trabecular meshwork cells after laser trabeculoplasty. Am J Ophthalmol..

