# Author Correction: The LRRC8-mediated volume-regulated anion channel is altered in glaucoma

**DOI:** 10.1038/s41598-020-68525-x

**Published:** 2020-07-07

**Authors:** Xavier Gasull, Marta Castany, Aida Castellanos, Mikel Rezola, Alba Andrés-Bilbé, Maria Isabel Canut, Raúl Estévez, Teresa Borrás, Núria Comes

**Affiliations:** 1 Neurophysiology Laboratory, Physiology Unit, Department of Biomedicine, Medical School, University of Barcelona, Casanova 143, 08036 Barcelona, Spain; 2 Institute of Neurosciences, University of Barcelona, Barcelona, Spain; 3 Institut d’Investigacions Biomèdiques August Pi i Sunyer (IDIBAPS), Barcelona, Spain; 4 Department of Ophthalmology, Hospital Vall d’Hebron, Passeig de la Vall d’Hebron 119-129, 08035 Barcelona, Spain; 5 Institut Universitari Barraquer, Muntaner 314, 08021 Barcelona, Spain; 6 Unitat de Fisiologia, Departament de Ciències Fisiològiques, University of Barcelona-IDIBELL, Centro de Investigación en Red de Enfermedades Raras (CIBERER), SCIII., Feixa Llarga S/N, L’Hospitalet de Llobregat, 08907 Barcelona, Spain; 7 Department of Ophthalmology, University of North Carolina, School of Medicine, 6109 Neuroscience Research Building, 105 Mason Farm Road, Chapel Hill, NC 27599-7041 USA

Correction to: *Scientific Reports* 10.1038/s41598-019-41524-3, published online 01 April 2019

The Acknowledgements section in this Article is incomplete.

“We are grateful to Dr. D.W. Stamer and his collaborators from Duke University Eye Center (Durham, N.C.) for their gift of human primary TM cells. The present work was supported by Ministerio de Economia y Competitividad and Instituto de Salud Carlos III of Spain FIS PI14/00141 (X.G.), FIS P17/00296 (X.G.), RETICs Oftared RD12/0034/0003 (X.G.), RD16/0008/0014 (X.G.), RD12/0034/0015 (M.C.), RD16/0008/0023 (M.C.), Generalitat de Catalunya 2014SGR1165 (X.G.), and National Eye Institute - NIH, EY-11906 (T.B.).”

should read:

“We are grateful to Dr. D.W. Stamer and his collaborators from Duke University Eye Center (Durham, N.C.) for their gift of human primary TM cells. The present work was supported by European Union, Fondo Europeo de Desarrollo Regional (FEDER), Ministerio de Economia y Competitividad and Instituto de Salud Carlos III of Spain FIS PI14/00141 (X.G.), FIS P17/00296 (X.G.), RETICs Oftared RD12/0034/0003 (X.G.), RD16/0008/0014 (X.G.), RD12/0034/0015 (M.C.), RD16/0008/0023 (M.C.), Generalitat de Catalunya 2014SGR1165 (X.G.), and National Eye Institute - NIH, EY-11906 (T.B.).”

