# Supplementary material for: The LRRC8-mediated volume-regulated anion channel is altered in glaucoma
Source: Sci Rep. 2019 Apr 1;9:5392. doi: 10.1038/s41598-019-41524-3 (PMC6443673; doi:10.1038/s41598-019-41524-3)
Supplement: Supplementary file 1 — Supplementary Information [file 41598_2019_41524_MOESM1_ESM.pdf]

The LRRC8-mediated volume-regulated anion channel is altered in glaucoma.

Xavier Gasull, Marta Castany, Aida Castellanos, Mikel Rezola, Alba Andrés-Bilbé, Maria Isabel Canut, Raúl Estévez, Teresa Borrás, Núria Comes.

(full-length blots)

Fig. 1

B

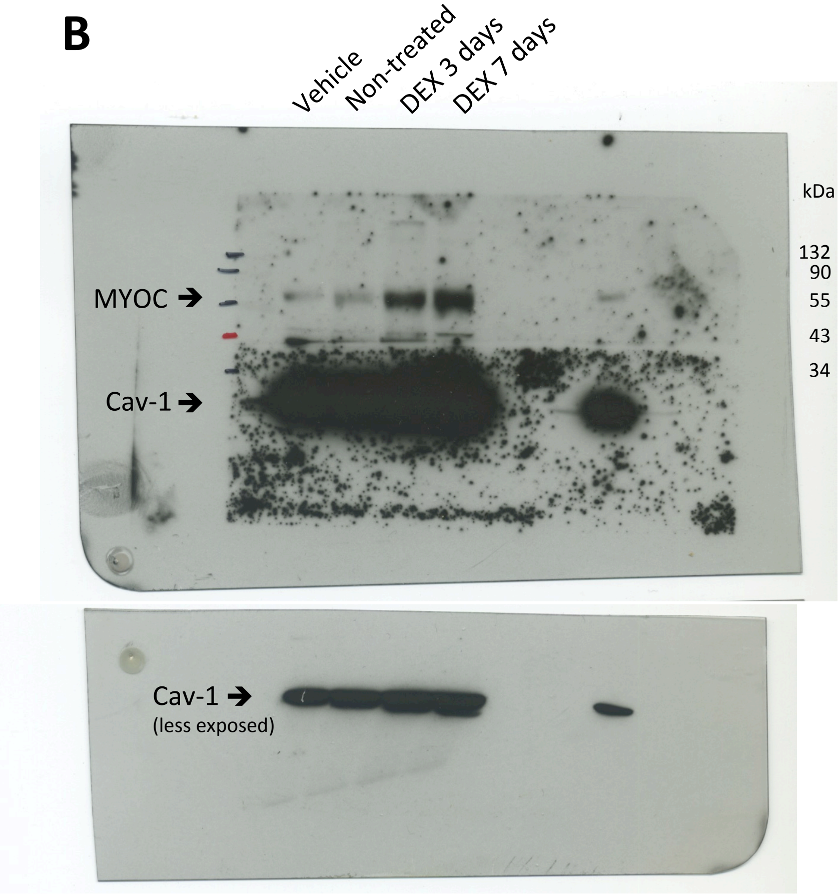
Vehicle
Non-treated
DEX 3 days
DEX 7 days
kDa
132
90
55
43
34
MYOC ➔
Cav-1 ➔
Cav-1 ➔
(less exposed)

Fig. 5

A

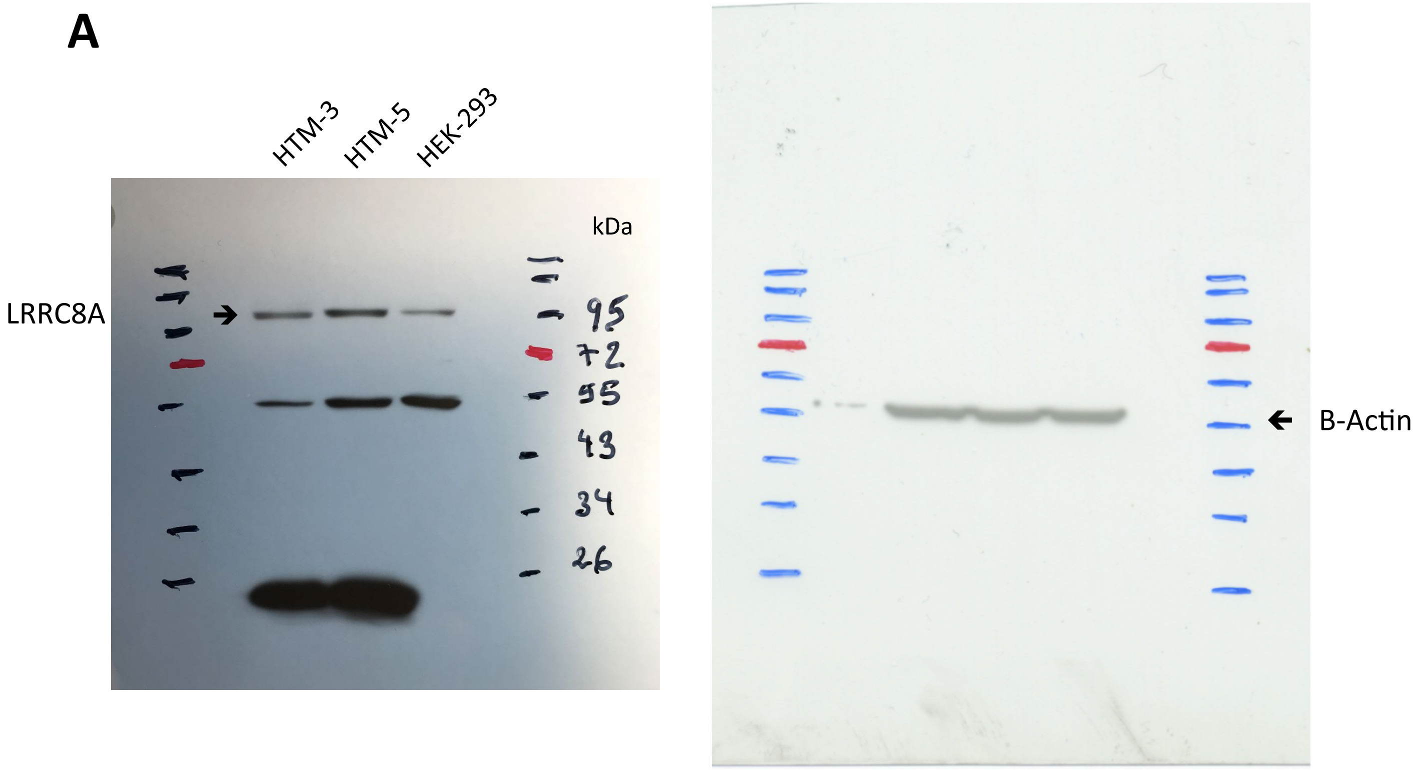
HTM-3
HTM-5
HEK-293
kDa
LRRC8A
95
72
55
43
34
26
B-Actin

B

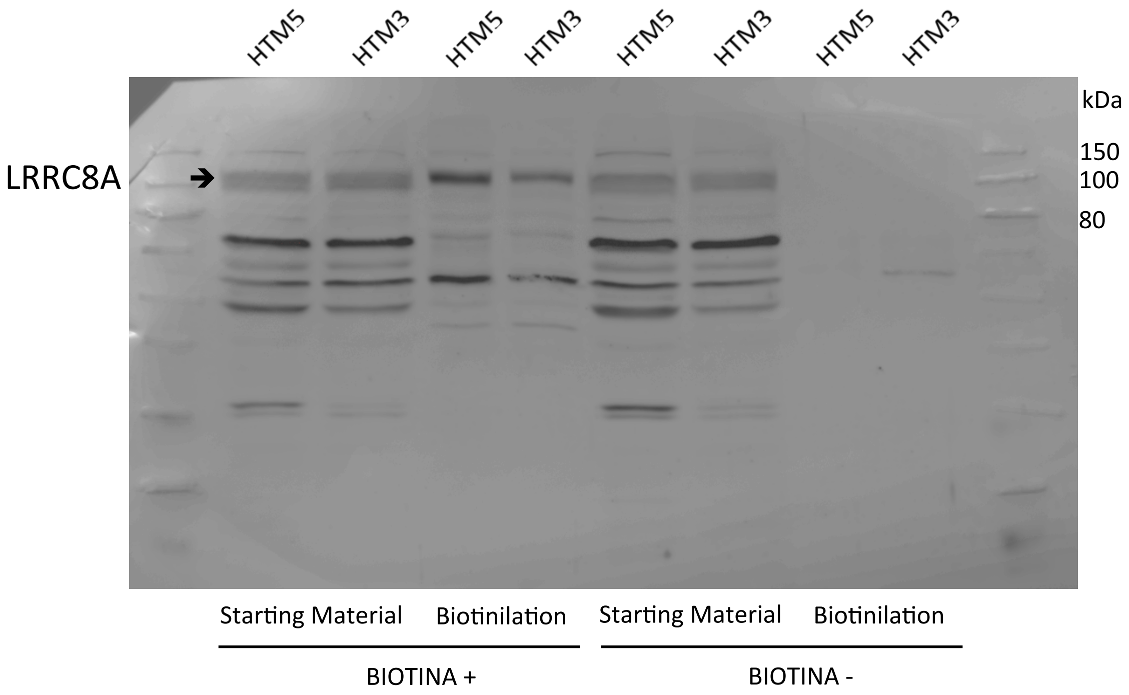
HTM5
HTM3
HTM5
HTM3
HTM5
HTM3
HTM5
HTM3
kDa
150
100
80
LRRC8A
Starting Material
Biotinilation
Starting Material
Biotinilation
BIOTINA +
BIOTINA -

Fig. 5

C

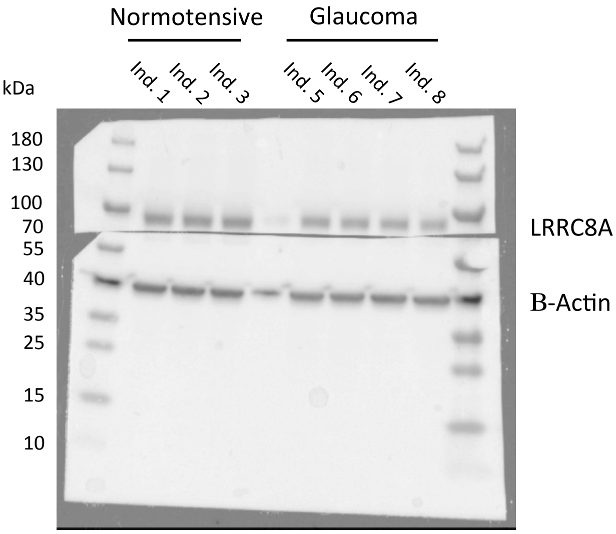
Normotensive
Glaucoma
Ind. 1
Ind. 2
Ind. 3
Ind. 5
Ind. 6
Ind. 7
Ind. 8
kDa
180
130
100
70
55
40
35
25
15
10
LRRC8A
B-Actin

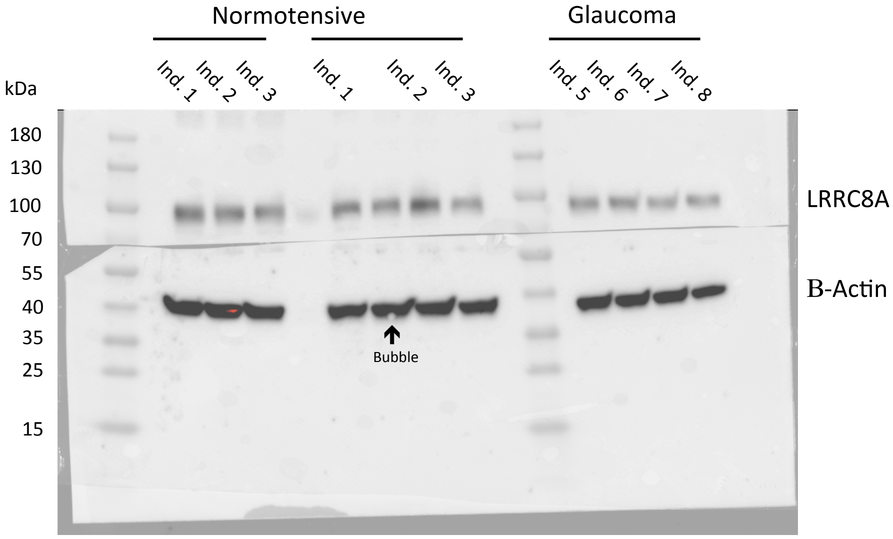
Normotensive
Glaucoma
Ind. 1
Ind. 2
Ind. 3
Ind. 1
Ind. 2
Ind. 3
Ind. 5
Ind. 6
Ind. 7
Ind. 8
kDa
180
130
100
70
55
40
35
25
15
LRRC8A
B-Actin
Bubble
